# Dynamic Immune Function Changes Before and After the First Radioactive Iodine Therapy After Total Resection of Differentiated Thyroid Carcinoma

**DOI:** 10.3389/fimmu.2022.901263

**Published:** 2022-06-30

**Authors:** Zhi-Yong Shi, Sheng-Xiao Zhang, Di Fan, Cai-Hong Li, Zhe-Hao Cheng, Yan Xue, Li-Xiang Wu, Ke-Yi Lu, Su-Yun Yang, Yan Cheng, Zhi-Fang Wu, Chong Gao, Xiao-Feng Li, Hai-Yan Liu, Si-Jin Li

**Affiliations:** ^1^Department of Nuclear Medicine, First Hospital of Shanxi Medical University, Taiyuan, China; ^2^Collaborative Innovation Center for Molecular Imaging of Precision Medicine, First Hospital of Shanxi Medical University, Taiyuan, China; ^3^Department of Rheumatology, Second Hospital of Shanxi Medical University, Taiyuan, China; ^4^Key Laboratory of Cellular Physiology, Ministry of Education, Shanxi Medical University, Taiyuan, China; ^5^Department of Pathology, Brigham and Women’s Hospital, Harvard Medical School, Boston, MA, United States

**Keywords:** regulatory T cells, CD4 + T cells, immune, differentiated thyroid carcinoma, radioactive iodine therapy

## Abstract

The effects of total thyroidectomy or radioactive iodine therapy on immune activation and suppression of the tumor microenvironment remain unknown. We aimed to investigate the effects of these treatments on the immune function in patients with differentiated thyroid carcinoma (DTC). Our cohort included 45 patients with DTC treated with total thyroidectomy and radioactive iodine therapy (RAIT). Immune function tests were performed by flow cytometry at 0, 30, and 90 days post-RAIT. Both the percentage and absolute number of circulating regulatory T cells were significantly lower in the postoperative DTC compared to the healthy controls. Notably, the absolute number of multiple lymphocyte subgroups significantly decreased at 30 days post-RAIT compared to those pre-RAIT. The absolute counts of these lymphocytes were recovered at 90 days post-RAIT, but not at pre-RAIT levels. Additionally, the Th17 cell percentage before RAIT was positively correlated with thyroglobulin (Tg) levels after RAIT. The tumor burden might contribute to increased levels of circulating Tregs. In conclusion, RAIT caused transient radiation damage in patients with DTC and the percentage of Th17 cells before RAIT could be a significant predictor of poor prognosis in patients with DTC.

First of all, we found that the tumor burden might contribute to increased levels of circulating Tregs, which may be the main reason for tumor immune tolerance. This will further confirm the important role of Tregs in the occurrence and development of tumor. Understanding the changes of Tregs after total thyroidectomy will clarify its role more clearly. Various positive clinical signs may cause changes in the absolute counts of different lymphocyte subsets, which may enable us to make more accurate and personalized treatment decisions. This will further guide the treatment of DTC patients. Most importantly, we found for the first time that RAIT caused transient radiation damage in DTC. This suggests that interventional therapy may be required to improve the prognosis of DTC patients. Understanding the immune effect of RAIT on DTC patients will have important guiding significance for clinical treatment in the future. However, whether the normalization of lymphocyte balance is beneficial to the patient remains unclear. Finally, the data suggest that the percentage of Th17 cells before RAIT could be a significant predictor of poor prognosis in patients with DTC.

## Introduction

Thyroid carcinoma is the most common malignant neoplasm of the endocrine system, and its incidence has been shown to be increasing worldwide, especially in younger patients (1–3). Differentiated thyroid carcinoma (DTC) accounts for 90% of all thyroid cancers (4) and although it is not life-threatening if treated appropriately, patients may die of thyroid cancer if it spreads to the lymph nodes or other organs.

Immune surveillance against cancer metastasis is important to protect the host against carcinogenesis, with immune escape acting as a key factor in poorer clinical outcomes in this kind of carcinoma and often negatively impacting therapeutic efficacy (5).

Recent studies have provided evidence that the occurrence and development of tumors is closely related to the immunosuppressive microenvironment (6, 7), especially the enhanced suppressive potential of regulatory T cells (Tregs) (8–10). However, the absolute changes in peripheral lymphocyte subsets (especially Th17 and Tregs) in patients with DTC remain unclear. Furthermore, thyroidectomy and postoperative radioactive iodine therapy (RAIT) are routinely used to treat DTC patients with potential metastatic disease (11, 12), but little is known about the effects of these treatments on the lymphocyte subsets in DTC patients.

Given the particular importance of changes in the immune system in cancer cell development and progression, as well as the potential role of the immune response in tumor recurrence, we designed this study to investigate the changes in circulating lymphocyte subsets in patients with DTC after total thyroidectomy, and their dynamic response to RAIT. The purpose of this study was to provide further insights into the effects of total thyroidectomy and RAIT on the immune function in DTC patients by means of a prospective clinical trial, and to provide a theoretical basis for anti-tumor immunotherapy clinical trials.

## Materials and Methods

### Patients

One hundred and five participants were recruited from First Hospital of Shanxi Medical University between January 2021 and August 2021, including 45 patients with DTC (27 women; 18 men; age 20–77 years) and 60 age- and sex-matched healthy controls (HC) (35 women; 25 men; age 26–70 years). All DTC patients were treated with thyroidectomy and RAIT, according to the EANM guidelines. Some laboratory indices and iodine doses before radiotherapy are shown in **Table S1**. LT4 was routinely administered following radioiodine therapy and patients with acute inflammatory or autoimmune diseases, chronic inflammatory or other cancers, or other diseases that might produce altered immunity were excluded from this study. None of the enrolled patients had been treated with glucocorticoids and/or immunosuppressive drugs within the previous 3 months.

Peripheral blood samples were collected from all patients and both thyroid and immune function tests were completed for each patient on day 0 before RAIT, and on days 30 and 90 after RAIT. Thyroid function tests included free T3, free T4, thyroid-stimulating hormone, antithyroglobulin antibody, and antithyroid peroxidase antibody (SN-697, Shanghai Nuclear Research Institute Rihuan Photoelectric Instrument Co., Ltd, China). This study was approved by the Ethics Committee of the First Hospital of Shanxi Medical University, and informed consent was obtained from all patients and controls.

### Flow Cytometry

**Figure 1:**
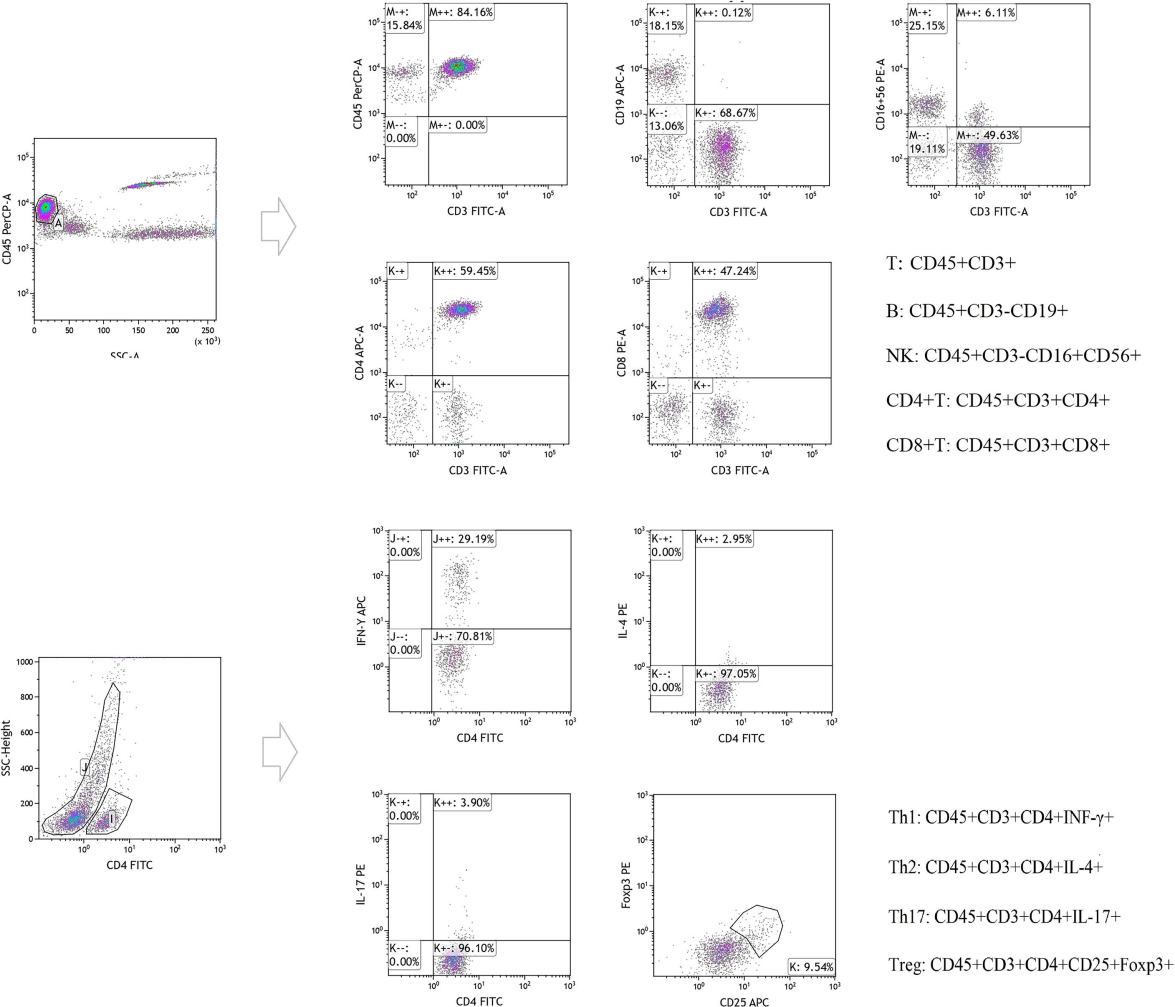
Original figure and cell-surface molecule markers of Lymphocyte subsets detected by flow cytometry.

The absolute number of lymphocytes in each of the various subpopulations isolated from the peripheral blood samples (CD3^+^ T/CD4^+^ T/CD8^+^ T/B/NK cells) were counted using flow cytometry. We first transferred 50 μL of EDTA-anticoagulated venous blood into A and B Trucount tubes (Becton-Dickinson, USA) and then the blood cells were collected using flow cytometry on a BD FACSCalibur and detected using the MultiSET software (BD Biosciences). The following conjugated monoclonal antibodies were used to identify the CD3+/CD4+/CD8+ T lymphocyte subsets: fluorescein isothiocyanate (FITC)-CD3, allophycocyanin (APC)-CD4, peridinin chlorophyll protein (PerpCP)-CD45, and phycoerythrin (PE)-CD8 (20210301, Beijing Tongsheng Shidai Biotech Co., Ltd, China). The following monoclonal antibodies were used for the B and NK cell populations: FITC-CD3, APC-CD19, PerpCPCD45, and PE-CD16+CD56 (20210726, Beijing Tongsheng Shidai Biotech Co., Ltd.). Then the CD4^+^ T cell subsets (Th1/Th2/Th17/Treg cells) were produced *via* the stimulation of Th1/Th2/Th17 cells using PMA and ionomycin. These cells were then labeled with FITC-conjugated anti-CD4 antibodies (20210604, Beijing Tongsheng Shidai Biotech Co., Ltd.), fixed, permeabilized using 1 mL freshly prepared fixation/permeabilization buffer, and then stained with APC-conjugated anti-IFN-γ (C7048040521503, Beijing Tongsheng Shidai Biotech Co., Ltd.)/PE-conjugated IL-4 (C7048040521503, Beijing Tongsheng Shidai Biotech Co., Ltd.)/PE-conjugated IL-17A antibodies (0314403, Beijing Tongsheng Shidai Biotech Co., Ltd.). Tregs were evaluated using cell surface-labeling with FITC-conjugated anti-CD4 and APC-conjugated anti-CD25 (0139110, Beijing Tongsheng Shidai Biotech Co., Ltd.), followed by fixation, permeabilization, and intracellular staining with PE-conjugated anti-Foxp3 antibody, in accordance with the manufacturer’s protocol. All antibodies were purchased from Beijing Tongsheng Shidai Biotechnology Co., Ltd. Then the percentage of each subpopulation was calculated using the CellQuest software version 6, and the absolute number of cells in each subpopulation was calculated as follows: absolute cell number = percentage of positive cells in each subgroup × absolute count of CD4^+^ T cells (cells/μL). For details, please see **Figure 1**.

### Statistical Analysis

All statistical analyses were performed using the SPSS22.0 and/or GraphPad Prism 6 software (GraphPad Software), and data are presented as the mean ± standard deviation (M ± SD) or median. Comparisons of results from each of the two groups were conducted using one-way analysis of variance, while the independent-samples were evaluated by Kruskal-Wallis test one-way analysis of variance. Correlation analysis was performed using the Pearson’s correlation test and the DTC patient data following total thyroidectomy were compared with healthy controls using an independent sample t-test or Mann-Whitney U test. Paired t-tests were used to compare pre-and post-treatment outcomes. Statistical significance was set at P < 0.05.

## Results

### Comparison of Clinical Data

The demographic and clinical data for our cohort are summarized in **Table 1**. Of the 45 subjects, 33 presented with clear invasion of the surrounding tissues (including nerve, muscle invasion, and blurring of boundaries with surroundings), 38 with lymphatic metastasis, 23 with tumors of more than 1 cm in size, 27 with unilateral foci, and 18 with single foci (**Table 1**).

**Table 1:** Characteristics of patients with DTC and HC.

	Patients	Healthy	P
N	45	60	–
Sex (women/man)	27/18	35/25	0.72
Surrounding tissue invasion	33/12	–	–
Size (>1cm)	23/22	–	–
Unilateral focus	27/18	–	–
Single focus	18/27	–	–
Lymphatic metastasis	38/7	–	–
Age (year), x ± s	43.0 ± 11.2	46.6 ± 14.1	0.166
FT3 (pmol/L), mean (range)	1.4 (0.5-3.3)	–	–
FT4 (pmol/L), mean (range)	1.7 (0.0-6.8)	–	–
TSH (μIU/mL), mean (range)	114.6 (43.7-150.0)	–	–
TgAb (U/mL), mean (range)	35.9 (15.0-172.2)	–	–
TPOAb (U/mL), mean (range)	87.1 (28.0-760.8)	–	–
Tg (ng/mL), mean (range)	10.6 (0.04-156.9)	–	–
WBC (10’9/L), mean (range)	5.6 (3.1-10.1)	–	–
Lymphocyte (10’9/L), mean (range)	1.6 (1.0-2.7)	–	–
Lymphocyte (%), mean (range)	29.8 (15.6-50.1)	–	–
Neutrophils (10’9/L), mean (range)	3.5 (1.5-7.9)	–	–
Neutrophils (%), mean (range)	62.1 (43.8-78.3)	–	–
Monocytes (10’9/L), mean (range)	0.2 (0.1-0.5)	–	–
Monocytes (%), mean (range)	4.6 (2.3-7.6)	–	–
ALT (U/L), mean (range)	37.4 (7.0-126.0)	–	–
AST (U/L), mean (range)	34.6 (13.0-95.0)	–	–
TC (mmol/L), mean (range)	7.2 (5.2-9.7)	–	–
TG (mmol/L), mean (range)	2.7 (0.9-8.6)	–	–
HDL (mmol/L), mean (range)	1.6 (1.1-2.2)	–	–
LDL (mmol/L), mean (range)	4.7 (3.3-6.7)	–	–

ALT, Alanine aminotransferase; AST, Aspartate aminotransferase; TC, Total Cholesterol; TG, Triglyceride; HDL, High density liptein cholesterol; LDL, Low density liptein cholesterol.

We noted that postoperative DTC patients with lymphatic metastasis also presented with an increased percentage of B cells and Tregs in their blood when compared with those without metastasis (P = 0.040, P = 0.044), while the percentage of CD4^+^ T cells and the absolute number of Th1 cells significantly decreased in these samples (P = 0.024, P = 0.037, **Tables 2**, **3**).

**Table 2:** Effect of clinical signs on lymphocyte subsets (T, B, NK, CD4^+^ T, and CD8^+^ T cells).

	T (cells/μL)	B (cells/μL)	NK (cells/μL)	CD4^+^ T(cells/μL)	CD8^+^ T(cells/μL)	T%	B%	NK%	CD4^+^ T%	CD8^+^ T%
**Age**										
≥50	1311.1 ± 350.7	247.6 ± 140.4	325.1 ± 133.9	652.1 ± 225.3	571.0 ± 298.1	68.1 ± 7.7	12.8 ± 6.3	17.3 ± 6.5	36.8 ± 8.3	28.9 ± 9.9
<50	1201.7 ± 344.4	232.9 ± 99.9	253.3 ± 117.9	664.0 ± 202.7	494.8 ± 163.3	70.3 ± 5.7	13.4 ± 4.1	14.3 ± 4.4	39.0 ± 5.6	28.8 ± 4.8
*P value*	0.342	0.694	0.082	0.863	0.276	0.313	0.716	0.076	0.319	0.966
**Gender**										
Male	1295.0 ± 441.1	235.7 ± 83.0	319.5 ± 158.0	680.0 ± 252.4	557.6 ± 278.8	68.1 ± 7.8	12.7 ± 3.9	16.8 ± 6.3	36.0 ± 6.9	29.0 ± 8.9
Female	1192.2 ± 266.1	238.1 ± 128.7	243.8 ± 89.3	647.6 ± 174.3	489.6 ± 148.8	70.6 ± 5.0	13.6 ± 5.3	14.1 ± 4.1	39.9 ± 5.8	28.7 ± 4.4
*P value*	0.334	0.945	0.046	0.612	0.293	0.195	0.53	0.08	0.049	0.867
**Size**										
>1	1221.4 ± 305.6	270.6 ± 126.9	311.7 ± 124.9	631.6 ± 184.7	503.3 ± 181.65	66.9 ± 5.3	14.6 ± 5.4	16.8 ± 5.6	36.3 ± 5.9	27.1 ± 5.1
≤1	1245.7 ± 390.5	202.1 ± 81.8	234.7 ± 116.2	690.9 ± 228.3	531.0 ± 240.3	72.5 ± 6.2	11.8 ± 3.6	13.5 ± 4.2	40.5 ± 6.5	30.7 ± 7.4
*P value*	0.817	0.038	0.038	0.342	0.664	0.002	0.053	0.035	0.032	0.069
**Lymphatic metastasis**										
Lymphatic metastasis	1227.0 ± 348.5	248.8 ± 113.1	271.3 ± 122.3	642.5 ± 203.5	520.8 ± 213.5	69.2 ± 6.6	13.8 ± 4.7	15.1 ± 5.5	37.4 ± 6.0	29.1 ± 6.9
Non-lymphatic metastasis	1267.3 ± 356.1	173.6 ± 82.7	289.1 ± 151.7	758.5 ± 212.8	495.4 ± 206.8	71.7 ± 4.6	9.9 ± 3.5	15.5 ± 3.0	43.4 ± 7.2	27.5 ± 3.7
*P value*	0.781	0.102	0.735	0.176	0.773	0.346	0.044	0.865	0.024	0.56
**Surrounding tissue invasion**										
Invasive group	1185.6 ± 293.3	230.0 ± 114.3	270.9 ± 134.3	654.4 ± 212.4	471.7 ± 145.1	69.5 ± 6.2	13.3 ± 5.1	15.3 ± 5.2	39.5 ± 6.2	27.7 ± 5.5
Non-invasive group	1350.7 ± 442.2	254.7 ± 106.9	281.8 ± 105.3	675.6 ± 200.2	627.9 ± 298.4	69.9 ± 6.8	13.0 ± 4.1	14.8 ± 5.2	35.6 ± 6.6	31.7 ± 8.1
*P value*	0.149	0.506	0.795	0.76	0.022	0.88	0.84	0.74	0.073	0.058
**Focus location**										
Unilateral focus	1229.4 ± 374.3	236.9 ± 98.3	285.5 ± 136.5	654.2 ± 250.3	517.9 ± 174.3	69.2 ± 5.9	13.3 ± 4.2	15.3 ± 4.5	38.1 ± 6.4	29.1 ± 4.6
Bilateral focus	1239.2 ± 308.7	237.4 ± 132.1	256.9 ± 108.6	670.1 ± 122.0	515.2 ± 260.9	70.2 ± 7.0	13.2 ± 5.6	15.0 ± 6.2	38.7 ± 6.7	28.4 ± 8.8
*P value*	0.927	0.988	0.461	0.804	0.968	0.603	0.937	0.839	0.796	0.736
**Lesion numbers**										
Single lesion	1142.0 ± 291.5	232.3 ± 99.4	270.2 ± 120.1	577.2 ± 171.5	510.2 ± 168.5	68.8 ± 4.7	13.7 ± 4.3	15.5 ± 4.2	37.1 ± 5.5	30.3 ± 3.5
Multiple lesions	1294.2 ± 370.5	240.3 ± 120.8	276.6 ± 131.3	716.2 ± 212.6	521.2 ± 237.5	70.2 ± 7.2	12.9 ± 5.1	14.9 ± 5.8	39.1 ± 7.0	27.9 ± 7.9
*P value*	0.15	0.815	0.868	0.026	0.866	0.456	0.564	0.704	0.319	0.233

**Table 3:** Effect of clinical signs on CD4^+^ T lymphocyte subsets (Th1, Th2, Th17 and Tregs).

	Th1 (cells/μL)	Th1 (cells/μL)	Th17 (cells/μL)	Treg (cells/μL)	Th1%	Th2%	Th17%	Treg%
**Age**								
**≥50**	179.3 ± 85.6	7.0 ± 3.2	9.3 ± 4.8	23.7 ± 8.1	27.0 ± 9.1	1.0 ± 0.3	1.4 ± 0.5	3.7 ± 0.6
**<50**	150.2 ± 74.6	7.4 ± 2.8	9.2 ± 3.5	26.0 ± 9.2	22.3 ± 8.4	1.1 ± 0.2	1.3 ± 0.3	3.9 ± 0.6
***P value***	0.263	0.738	0.939	0.434	0.11	0.728	0.879	0.311
**Gender**								
**Male**	170.0 ± 84.8	7.3 ± 3.3	10.3 ± 4.4	26.8 ± 10.3	24.6 ± 7.0	1.1 ± 0.3	1.5 ± 0.4	3.9 ± 0.6
**Female**	151.0 ± 73.9	7.2 ± 2.7	8.5 ± 3.4	24.3 ± 7.8	23.0 ± 9.8	1.1 ± 0.2	1.3 ± 0.3	3.7 ± 0.6
***P value***	0.432	0.927	0.122	0.355	0.571	0.756	0.051	0.326
**Size**								
**>1**	147.9 ± 74.5	7.2 ± 2.9	8.5 ± 3.8	24.3 ± 8.5	23.3 ± 10.0	1.1 ± 0.2	1.3 ± 0.4	3.8 ± 0.6
**≤1**	169.8 ± 81.8	7. 3± 3.0	10.0 ± 3.8	26.3 ± 9.3	24.1 ± 7.5	1.0 ± 0.3	1.4 ± 0.3	3.8 ± 0.6
***P value***	0.355	0.918	0.193	0.455	0.771	0.51	0.28	0.994
**Lymphatic metastasis**								
**Lymphatic metastasis**	148.3 ± 71.8	7.05 ± 3.0	9.1 ± 4.0	25.3 ± 9.1	23.0 ± 8.8	1.1 ± 0.3	1.3 ± 0.4	3.9 ± 0.6
**Non-lymphatic metastasis**	214.7 ± 92.3	8.7 ± 1.6	10.0 ± 3.0	25.4 ± 8.0	27.1 ± 8.0	1.2 ± 0.3	1.3 ± 0.3	3.4 ± 0.7
***P value***	0.037	0.161	0.585	0.981	0.26	0.374	0.875	0.04
**Surrounding tissue invasion**								
**Invasive group**	153.8 ± 83.4	7.6 ± 3.1	9.0 ± 4.0	24.7 ± 8.6	22.8 ± 9.1	1.1 ± 0.3	1.3 ± 0.4	3.8 ± 0.6
**Non-invasive group**	170.4 ± 64.6	6.5 ± 2.4	9.7 ± 3.7	26.9 ± 9.6	25.7 ± 7.7	1.0 ± 0.2	1.4 ± 0.3	3.9 ± 0.5
***P value***	0.526	0.288	0.587	0.463	0.323	0.103	0.574	0.565
**Focus location**								
**Unilateral focus**	156.3 ± 84.8	7.4 ± 3.2	9.0 ± 4.2	26.0 ± 10.7	23.2 ± 8.1	1.1 ± 0.2	1.3 ± 0.3	3.9 ± 0.6
**Bilateral focus**	162.1 ± 69.0	7.0 ± 2.5	9.5 ± 3.4	24.3 ± 5.3	24.3 ± 9.9	1.0 ± 0.3	1.4 ± 0.4	3.6 ± 0.6
***P value***	0.808	0.665	0.699	0.557	0.682	0.32	0.638	0.12
**Lession numbers**								
**Single lesion**	143.0 ± 79.1	6.9 ± 2.2	7.5 ± 2.7	23.0 ± 8.2	23.7 ± 9.3	1.2 ± 0.2	1.3 ± 0.3	3.9 ± 0.6
**Multiple lesions**	169.0 ± 77.1	7.5 ± 3.3	10.4 ± 4.2	26.9 ± 9.1	23.6 ± 8.6	1.0 ± 0.3	1.4 ± 0.4	3.7 ± 0.6
***P value***	0.278	0.495	0.014	0.15	0.96	0.05	0.228	0.349

In addition, when we compared patients with tumors of up to 1 cm in size with those with larger tumors we noted that the percentages of total T, CD4^+^ T and NK cells in the peripheral blood were significantly different (P = 0.002, P = 0.032, P = 0.035, as shown in **Table 2**). However, lymphocyte subsets in postoperative DTC patients showed no significant difference in absolute T and CD4^+^ T cell counts between the same two groups (P > 0.05, **Table 2**), while the absolute number of both B and NK cells significantly increased (P = 0.038 and P = 0.038, respectively; **Table 2**).

In addition, there was no significant change in the percentage of peripheral lymphocyte subsets in postoperative DTC patients with single or multiple lesions (P > 0.05, **Tables 2**, **3**). Similar results were also observed in postoperative DTC patients with and without invasion of the surrounding tissues (P > 0.05, **Tables 2**, **3**). However, improved flow cytometry analysis showed that the absolute number of Th17 cells in the peripheral blood of postoperative DTC patients with multiple lesions increased significantly (P = 0.014, **Table 3**) and led to an increase in the absolute number of CD4^+^ T cells (P = 0.026, **Table 2**). The absolute number of CD8^+^ T cells decreased significantly in the peripheral blood of postoperative DTC patients with invasion of the surrounding tissues (P = 0.02, **Table 2**) and the percentage of CD4^+^ T cells in the peripheral blood of male patients decreased (P = 0.049, **Table 2**), while the absolute number of NK cells increased significantly (P = 0.046, **Table 2**) compared to female samples. Age and lesion focus had no effect on the immune populations in the patients’ peripheral blood (P > 0.05; **Tables 2**, **3**). This study shows that the lymphocyte balance in these patients is affected by a particular clinical phenotype.

### Comparative Analysis of Lymphocyte Subsets in the Peripheral Blood of Postoperative DTC Patients and HC

**Figure 2:**
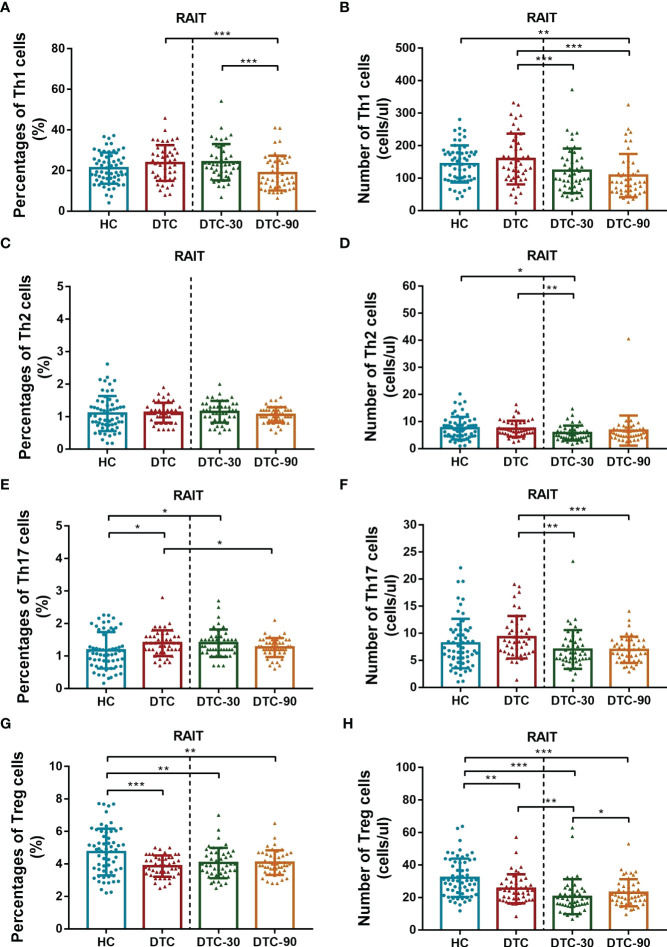
Comparison of levels of CD4^+^ T subsets (Th1, Th2, Th17 and Tregs) between postoperative DTC patients (pro- and post-RAIT) and HC. **(A, C, E, G)**: Percentage of CD4^+^ T cell subsets; **(B, D, F, H)**: Absolute counts of CD4^+^ T cell subsets. DTC, differentiated thyroid cancer; Tregs, regulatory T cells; RAIT, radioiodine treatment; DTC-30, 30 days after RAIT; DTC-90, 90 days after RAIT. *p < 0.05, **p < 0.01, ***p < 0.001. Asymptotic significances (2-sided tests) were displayed. The significance level was p < 0.05.

The absolute number and frequencies of the lymphocyte subsets were assessed using advanced flow cytometry. These evaluations revealed that the percentage of Th17 cells in the peripheral blood of postoperative DTC patients increased significantly (P = 0.027, **Figure 2E**), while the percentage of Tregs decreased significantly (P < 0.001, **Figure 2G**) in comparison with the HC. Interestingly, we found that the absolute number of Tregs in postoperative DTC patients decreased significantly (P = 0.002, **Figure 2H**), while the absolute number of Th17 cells did not change significantly when compared to the control (P = 0.172, **Figure 2F**). No significant differences were observed in the percentage or absolute number of total T, total B, CD4^+^ T, CD8^+^ T, NK, Th1, or Th2 cells between the patient and HC samples (P > 0.05, **Figure 2**). Taken together, these results suggest that Tregs play a critical role in the mechanism underlying immune dysregulation in patients with DTC.

### Dynamic Changes in the Peripheral Blood Lymphocyte Subsets of Patients With DTC Before and After RAIT

**Figure 3:**
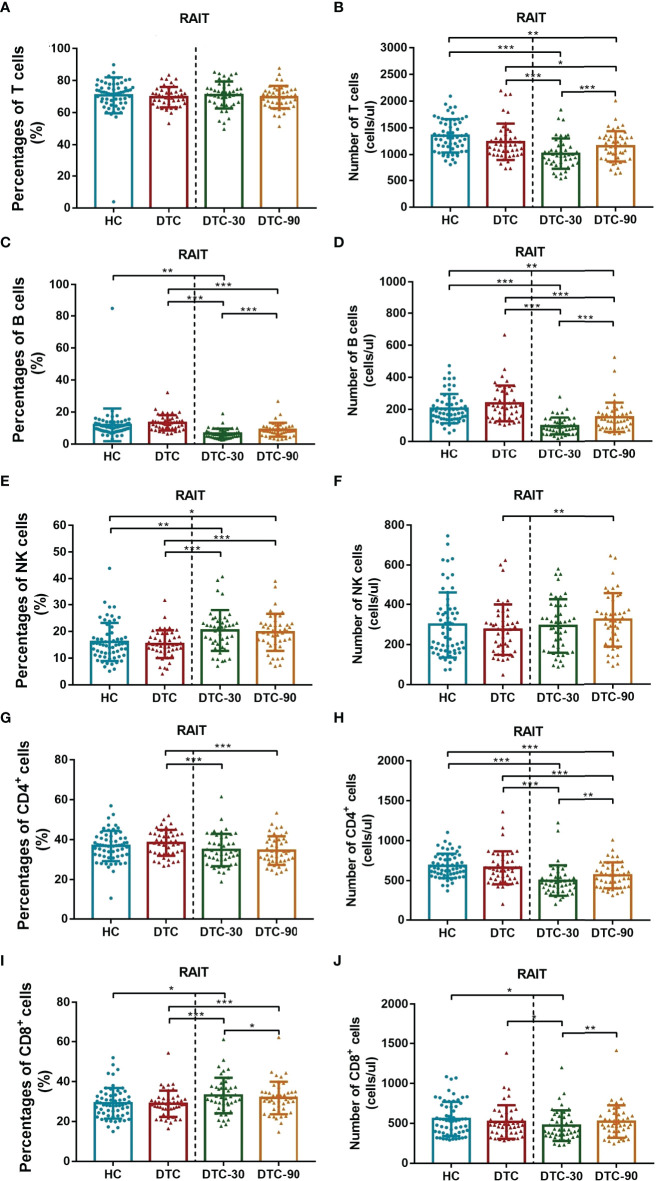
Comparison of levels of peripheral lymphocyte subset (T, B, NK, CD4^+^ T and CD8^+^ T cells) between postoperative DTC patients (pro- and post-RAIT) and HC. **(A, C, E, G, I)**: Percentage of peripheral lymphocyte subset; **(B, D, F, H, J)**: Absolute counts of peripheral lymphocyte subset. DTC, differentiated thyroid cancer; Tregs, regulatory T cells; RAIT, radioiodine treatment; DTC-30, 30 days after RAIT; DTC-90, 90 days after RAIT. *p < 0.05, **p < 0.01, ***p < 0.001. Asymptotic significances (2-sided tests) were displayed. The significance level was p < 0.05.

Given our previous findings, we then went on to determine whether RAIT might have some influence on peripheral lymphocyte subset distribution using advanced flow cytometry. We evaluated the absolute number and percentage of peripheral lymphocyte subsets on day 0 (before RAIT), day 30, and day 90 post RAIT. These evaluations showed that the percentage of total B (P < 0.001, **Figure 3C**) and CD4^+^ T cells (P < 0.001, **Figure 3G**) decreased significantly, while the percentage of total CD8^+^ T (P < 0.001, **Figure 3I**), and NK cells (P < 0.001, **Figure 3E**) increased significantly 30 days after RAIT when compared to the pre-RAIT values. On day 90, the percentage of total CD8^+^ T cells (P < 0.05, **Figure 3I**) decreased significantly compared to day 30 post RAIT, while the percentage of total B cells (P < 0.001, **Figure 3C**) increased significantly. However, the percentages of total T, CD4^+^ T, and NK cells did not change significantly (both P > 0.05, **Figure 3**). There were significant differences in total B, NK, CD4^+^ T, and CD8^+^ T cells 90 days after RAIT compared with pre-RAIT levels (both P < 0.001, **Figure 3**). In addition, on day 30, the patients’s circulating CD4^+^ T cell subsets showed no significant changes compared with those before RAIT (all P > 0.05, **Figure 2**). At 90 days after RAIT, the percentage of only circulating Th1 cells (P < 0.001, **Figure 2A**) decreased significantly compared to 30 days after RAIT. However, the impact of RAIT on immune function has not been fully elucidated by this analysis, thus we used improved flow cytometry to determine the absolute count for each of these lymphocyte subsets before and after RAIT in DTC patients. These data revealed that the absolute count for all of the lymphocyte subsets except NK were reduced significantly 30 days after RAIT (both P < 0.05, **Figures 2**, **3**). This is consistent with the suggestion that RAIT decreases immune function as a whole in patients who receive treatment. At day 90, the absolute counts of total T (P < 0.001), total B (P < 0.001), CD4^+^ T (P < 0.01), CD8^+^ T (P < 0.01), and Tregs (P < 0.05) increased significantly compared to day 30 post-RAIT, but most (total T, total B, and CD4^+^ T) did not reach pre-RAIT levels (P < 0.05, **Figures 2**, **3**). Furthermore, after 90 days, the absolute counts of circulating Th1 and Th17 cells were much lower (both P < 0.001, **Figures 2B, F**), but NK cell absolute counts (P < 0.01, **Figure 3F**) were significantly greater than before iodine therapy. Significant decreases in Th1 and Th17 cells may be linked to poor patient outcomes, but increases in absolute NK cell numbers are linked to the body’s response to microbial infection. This could be due to the body’s response to microbial infections. Taken together, these results suggest that the frequency of lymphocyte subsets does not fully reflect the immune status of patients with DTC.

### Balance of Lymphocyte Subsets Pre- and Post-RAIT

**Figure 4:**
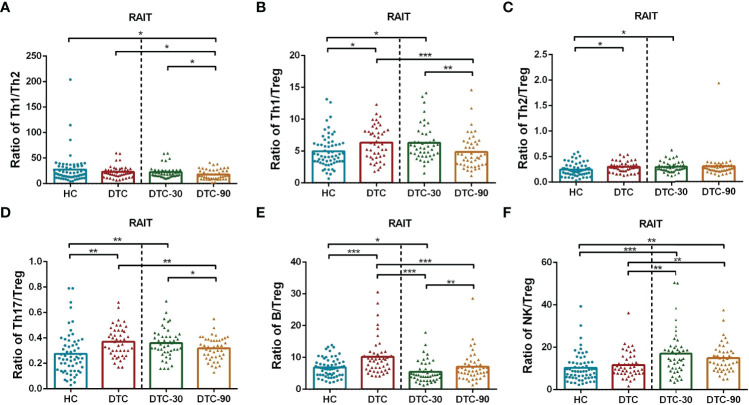
Comparison of ratios of lymphocyte subsets in postoperative DTC patients (pro- and post-RAIT) and HC. **(A–F)**: Ratio of each lymphocyte subsets. DTC, differentiated thyroid cancer; Tregs, regulatory T cells; RAIT, radioiodine treatment; DTC-30, 30 days after RAIT; DTC-90, 90 days after RAIT. *p < 0.05, **p < 0.01, ***P < 0.001. Asymptotic significances (2-sided tests) were displayed. The significance level was p < 0.05.

The Th1/Tregs, Th2/Tregs, Th17/Tregs, and B/Tregs ratios were significantly increased in patients with DTC at day 0 when compared to those of the HC (P < 0.05, **Figures 4B–E**). However, no significant differences were observed in the Th1/Th2 and NK/Treg ratios between these two groups (P > 0.05; **Figures 4A, F**). At day 30 post-RAIT, the B/Treg ratio were remarkably reduced, and the NK/Treg ratio increased significantly when compared to the pre-RAIT values (both P < 0.05, **Figure 4**). However, there were no significant changes in the Th1/Th2, Th1/Tregs, Th2/Tregs and Th17/Tregs ratios (P > 0.05, **Figures 4A–D**). On day 90, the ratios of Th1/Tregs, Th1/Th2, and Th17/Tregs decreased significantly(P < 0.05, **Figures 4A, B, D**), but there were no significant changes in the Th2/Tregs and NK/Tregs ratios (P > 0.05, **Figures 4C, F**). The B/Tregs ratio was restored when compared to day 30 post-RAIT (P < 0.01, **Figure 4E**), but not to pre-RAIT levels (P < 0.001, **Figure 4E**). In addition, the Th1/Tregs, T2/Tregs, Th17/Tregs, B/Tregs, and NK/Tregs ratios were all significantly different at 30 days post-RAIT when compared to the HC, but there was no significant difference in Th1/Th2. There was no significant difference in the Th1/Tregs, T2/Tregs, Th17/Tregs, and B cell/Tregs ratios from the 90-day post-RAIT and HC samples (both P > 0.05, **Figures 4B–E**), while the Th1/Th2 ratio decreased significantly (P < 0.05, **Figure 4A**) and the NK/Tregs ratio increased significantly (P < 0.01, **Figure 4F**) in these comparisons. This suggests that RAIT partially recovers immune balance by reducing the absolute count of lymphocyte subsets to varying degrees, but its potential effect on prognosis is not clear.

### Correlation Between Peripheral Blood Lymphocyte Subsets and Thyroglobulin (Tg) Levels in DTC Patients

Finally, we went on to evaluate the correlation between peripheral blood lymphocyte subsets and Tg levels. These evaluations revealed a significant correlation between Th17 cell counts before RAIT and Tg levels 30 days after RAIT (P = 0.03, **Table 4**), but there were no other correlations between the other lymphocyte subsets before RAIT and Tg levels at 30 days after RAIT (**Table 4**). The same trend was observed for the Th17 percentage before RAIT and Tg levels on day 90 after RAIT (P = 0.004, **Table 5**). In addition, there were no significant associations between lymphocyte subsets and Tg levels at the same stage (**Tables S2**, **3**).

**Table 4:** Correlation between lymphocyte subsets before RAIT and Tg at day 30 after RAIT.

Percentage	Spearman r	P value	Absolute numbers	Spearman r	P value
T%	-0.254	0.092	T (cells/µL)	0.101	0.511
B%	0.181	0.234	B (cells/µL)	0.246	0.104
NK%	0.175	0.252	NK (cells/µL)	0.192	0.205
CD4^+^ T%	-0.152	0.318	CD4^+^ T (cells/µL)	0.087	0.571
CD8^+^ T%	-0.091	0.552	CD8^+^ T(cells/µL)	0.086	0.575
Th1%	0.085	0.580	Th1 (cells/µL)	0.115	0.451
Th2%	0.075	0.624	Th2 (cells/µL)	0.100	0.514
Th17%	0.323	0.030^*^	Th17 (cells/µL)	0.276	0.066
Treg%	0.030	0.845	Treg (cells/µL)	0.099	0.517

*p < 0.05.

**Table 5:** Correlation between lymphocyte subsets before RAIT and Tg at day 90 after RAIT.

Percentage	Spearman r	P value	Absolute numbers	Spearman r	P value
T%	-0.337	0.024^*^	T (cells/µL)	-0.054	0.725
B%	0.135	0.376	B (cells/µL)	0.148	0.333
NK%	0.318	0.033^*^	NK (cells/µL)	0.255	0.091
CD4^+^ T%	-0.099	0.516	CD4^+^ T (cells/µL)	0.021	0.891
CD8^+^ T%	-0.235	0.120	CD8^+^ T (cells/µL)	-0.094	0.537
Th1%	0.094	0.538	Th1 (cells/µL)	0.082	0.592
Th2%	0.092	0.549	Th2 (cells/µL)	0.073	0.633
Th17%	0.426	0.004^*^	Th17 (cells/µL)	0.284	0.059
Treg%	0.054	0.724	Treg (cells/µL)	0.046	0.762

*p < 0.05.

## Discussion

Tregs can be induced and differentiated within the tumor microenvironment by a variety of naive T cells, which exert a strong immunosuppressive function, inhibit antitumor immunity, and promote the occurrence and development of tumors (13). Tregs can suppress the function of immune effector cells, including Th17 cells, through a variety of mechanisms, and are key factors in tumor immune escape. A loss of balance between Th17 cells and Tregs is thought to be the primary mechanism of this escape (14, 15). Previous studies have shown that the proportion of Tregs in the peripheral blood is significantly increased in patients with malignant tumors (16–19). The same trend was observed in response to tumor infiltration and the microenvironment of several cancers (20, 21), including thyroid cancer (8, 22, 23).

Although the absolute number of Tregs and other lymphocyte subsets also play key roles in immune homeostasis, their changes in DTC are almost totally unknown. We have emphasized the necessity of detecting the absolute numbers of circulating lymphocyte subsets in clinical laboratory examinations (24). In addition, we did not fully understand the immune status of DTC patients following total thyroidectomy, however, this has been largely addressed here *via* our application of modified flow cytometry.

Our data is the first to show that only the number of circulating Tregs were significantly reduced in DTC patients following total thyroidectomy when compared to HC, while the number of Th17 cells did not change significantly. This reduction should result in reduced immunosuppression and immune escape in the remaining tumor tissues.

Next, we analyzed the association between lymphocyte subsets and the general clinical characteristics of postoperative DTC patients. We showed that lymphatic metastasis had a strong association with Th1 cell numbers, while tumor volume was found to be associated with both B and NK cell counts. In addition, the number of foci was shown to be related to the absolute Th17 and CD4^+^ T cell count while the presence of surrounding invasion was highly correlated with the absolute number of CD8^+^ T cells. We also showed that there was a relationship between sex and absolute NK cell count. Additionally, age and whether the lesion was restricted to one side did not affect the immune status of the postoperative DTC patients. Thus, this data suggests that changes in the absolute numbers of these lymphocyte subsets were significantly correlated with lymphatic metastasis, tumor volume, number of lesions, infiltration of surrounding tissue, and sex differences. In the future, a better understanding of the underlying mechanisms of immune differences in different DTC patients will further refine risk classification, and may also direct us to more effective, individualized treatment strategies.

In general, DTC patients are treated with RAIT following total thyroidectomy in order to facilitate the best clinical outcome (25). However, we do not fully understand the effect of RAIT on circulating lymphocytes and the dynamic changes in these subgroups before and after RAIT, particularly in terms of their absolute number. Our results showed that the absolute numbers of multiple lymphocyte subsets were significantly reduced and changed in response to RAIT, suggesting that RAIT causes radiation damage to these cells. However, most of these peripheral lymphocyte subsets are largely recovered 90 days post RAIT although, none were restored to pre-RAIT levels. Our findings show that there was no significant difference in circulating Th1, Th2, or Th17 cells in DTC patients on the 90th day after RAIT compared to the 30th day after RAIT for CD4^+^ T cell subsets. However,we have observed that Th1 cells are still in a downward trend, which might be a reason in certain DTC patients’ poor prognosis. Th1 cells have been proven to play a critical function in anti-tumor immunity (26, 27). Another study demonstrated that the Th2 cytokines decreased following RAIT, but that there were no significant changes in either Th1 or Th17 associated cytokines (28). The short observation period could be a major reason for these observations. We can confidently state that this paper is the first to systematically reveal that RAIT can cause comprehensive damage to the immune system of patients with postoperative DTC. We also revealed that immune function gradually recovered over time.

When analyzing the change in the ratio of lymphocyte subsets, we found that the balance of lymphocyte subsets in postoperative DTC patients was dysregulated. Similar dysregulation of Th1/Th2 and Th17/Tregs has also been observed in patients with cervical cancer (29). Additionally, our study suggests that part of the immune balance returns to normal after 90 days post-RAIT, although it is not clear whether this is beneficial for patient prognosis. In addition, our data reveals a significant positive correlation between the percentage of Th17 cells before iodine therapy and TG after iodine therapy. This suggests that the percentage of Th17 cells before RAIT could be a significant predictor of poor prognosis in DTC patients.

Previous studies have indicated that autoimmune disease may be a predictive indicator of better survival and prognosis in cancer (30–32). Several animal experiments have further demonstrated that depletion of Tregs often improves anti-tumor immune responses (33, 34). These results suggest that immunosuppression caused by the tumor microenvironment is reversible. This provides theoretical guidance for the application of immunotherapy in DTC patients, such as specific Treg depletion (35). However, another study revealed that chronic lymphocytic thyroiditis cannot be used as an independent prognostic factor for thyroid carcinoma and is not associated with a lower relapse rate or distant metastasis (36). Consequently, further in-depth studies are required (37, 38).

This study has two limitations. First, the sample size is relatively small and second, whether the immune system damage caused by RAIT can return to pre-trauma levels or even above these levels has not been established. This may provide greater insight into the biological mechanisms of recovery. Therefore, larger, prospective, controlled studies of longer duration are needed to determine the long-term effects of RAIT on the prognosis of DTC patients.

In summary, we found that both the absolute number and percentage of circulating Tregs in DTC patients were significantly decreased after total thyroidectomy, suggesting that the tumor burden might contribute to increased levels of circulating Tregs. Increased Tregs may be the main reason for tumor immune tolerance. Additionally, RAIT resulted in a dramatic and transient reduction of multiple lymphocyte subsets, indicating radiation damage to the immune system in patients with DTC. In such a setting, the population of DTC patients may benefit from therapies to restore immune function after RAIT; the earlier the immune function recovers, the greater the benefit to the patient. All in all, this study elucidates the effects of total thyroidectomy and RAIT on the immune responses in DTC patients. This provides a direction for future clinical investigations on cancer immunotherapy. In their investigation of dedifferentiated liposarcoma, Schroeder et al. observed that patients with effective therapy had fewer circulating CD4^+^ T cell subsets than patients with poor efficacy (39). Low clonal CD4^+^ T cell subsets are related with higher treatment success, according to this study. As a result, investigating the dynamic differences in circulating immune cells in DTC patients with varying treatment results before and after RAIT will be more useful in developing focused and accurate treatment regimens and improving prognosis.

Various positive clinical signs may cause changes in the absolute counts of different lymphocyte subsets, which may enable us to make more accurate and personalized treatment decisions. Moreover, the tumor burden might contribute to increased levels of circulating Tregs, which may be the main reason for tumor immune tolerance. Most importantly, we found for the first time that RAIT caused transient radiation damage in DTC. Inadditionally, significant decreases in Th1 and Th17 cells may be linked to poor patient outcomes, but increases in absolute NK cell numbers are linked to the body’s response to microbial infection. This dynamic shift in circulating CD4^+^ T cell subsets before and after RAIT appears to be more favorable to identifying DTC individuals with different prognoses and then studying the immunological mechanism behind this difference. Whether the normalization of lymphocyte balance is beneficial to the patient remains unclear. Finally, the data suggest that the percentage of Th17 cells before RAIT could be a significant predictor of poor prognosis in patients with DTC.

## Data Availability Statement

The original contributions presented in the study are included in the article/**Supplementary Material**. Further inquiries can be directed to the corresponding authors.

## Ethics Statement

The studies involving human participants were reviewed and approved by the institutional review board of The First Hospital of Shanxi Medical University. The patients/participants provided their written informed consent to participate in this study.

## Author Contributions

All authors contributed to data acquisition. Z-YS and S-XZ provided the study design. C-HL, Z-HC, DF, and YX performed the sample collection and completed the patient communication. Z-FW, L-XW, K-YL, S-YY, and YC contributed to the discussion. Z-YS analyzed the data and wrote the manuscript. H-YL, S-JL, CG, and X-FL critically revised the manuscript. All authors were responsible for data analysis and manuscript preparation and approve its submission to this journal.

## Funding

This work was supported by grants from the National Natural Science Foundation of China (No. 82001740), the Ministry of Education of the People’s Republic of China, Personnel and Social Affairs Department of Shanxi Province (202003), Department of Finance of Shanxi Province (2019023), and the Science and Technology Department of Shanxi Province (201903D321194, 201804D131042).

## Conflict of Interest

The authors declare that the research was conducted in the absence of any commercial or financial relationships that could be construed as a potential conflict of interest.

## Publisher’s Note

All claims expressed in this article are solely those of the authors and do not necessarily represent those of their affiliated organizations, or those of the publisher, the editors and the reviewers. Any product that may be evaluated in this article, or claim that may be made by its manufacturer, is not guaranteed or endorsed by the publisher.

## Abbreviations

APC, allophycocyanin; DTC, differentiated thyroid carcinoma; FITC, fluorescein isothiocyanate; HC, healthy control; PE, phycoerythrin; RAIT, radioactive iodine therapy; Treg, regulatory T cell.
