# Supplementary material for: Dynamic Immune Function Changes Before and After the First Radioactive Iodine Therapy After Total Resection of Differentiated Thyroid Carcinoma
Source: Front Immunol. 2022 Jun 30;13:901263. doi: 10.3389/fimmu.2022.901263 (PMC9280633; doi:10.3389/fimmu.2022.901263)
Supplement: Supplementary file 1 [file DataSheet_1.docx]

Supplementary Material

# Supplementary Tables

**
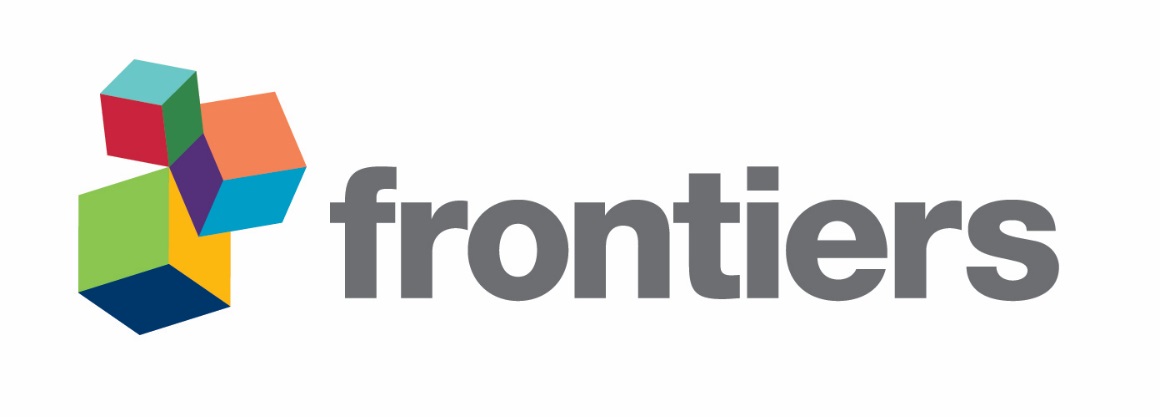
**

## Supplementary Tables

| Table S1. Some laboratory indexes and iodine dose before RAIT in patients with DTC | | | | |
| --- | --- | --- | --- | --- |
| SN | TSH level before RAIT (μIU/ml) | Tg level before RAIT (mmol/L) | UI before RAIT (μg/L) | Iodine dose **(mci)** |
| 1 | 76.73 | 3.63 | 218.8 | 100 |
| 2 | >150 | 1.93 | 101.50 | 100 |
| 3 | ＞150 | 38.56 | 83.20 | 150 |
| 4 | 118.6 | 156.90 | 88.00 | 150 |
| 5 | ＞150 | 0.04 | 191.60 | 100 |
| 6 | 138.77 | 1.68 | 166.30 | 100 |
| 7 | 103.79 | 0.21 | 104.90 | 100 |
| 8 | >150 | 0.04 | 280.80 | 125 |
| 9 | 54.51 | 0.78 | 51.50 | 100 |
| 10 | >150 | 0.07 | 373.30 | 100 |
| 11 | 118.26 | 1.00 | 88.80 | 100 |
| 12 | >150 | 3.60 | 95.80 | 125 |
| 13 | 77.17 | 0.04 | 144.40 | 100 |
| 14 | >150 | 0.93 | 318.10 | 100 |
| 15 | 90.894 | 0.87 | 244.00 | 100 |
| 16 | 138.92 | 23.12 | 200.90 | 125 |
| 17 | 143.96 | 14.83 | 86.40 | 100 |
| 18 | 76.727 | 17.82 | 152.10 | 100 |
| 19 | 114.13 | 34.07 | 134.10 | 125 |
| 20 | >150 | 5.82 | 157.00 | 100 |
| 21 | 136.55 | 2.48 | 146.20 | 100 |
| 22 | 104.17 | 2.77 | 246.00 | 150 |
| 23 | 127.29 | 45.25 | 262.20 | 150 |
| 24 | 96.42 | 0.75 | 159.90 | 100 |
| 25 | 117.86 | 8.20 | 554.50 | 100 |
| 26 | 123.83 | 0.04 | 0.10 | 100 |
| 27 | >150 | 26.74 | 329.40 | 100 |
| 28 | >150 | 0.04 | 57.60 | 100 |
| 29 | 64.32 | 4.91 | 117.30 | 100 |
| 30 | 137.71 | 17.64 | 153.80 | 100 |
| 31 | >150 | 0.04 | 131.70 | 100 |
| 32 | 47.96 | 3.76 | 209.10 | 100 |
| 33 | >150 | 3.16 | 57.40 | 100 |
| 34 | 109.83 | 1.11 | 61.00 | 100 |
| 35 | 43.73 | 1.18 | 738.90 | 100 |
| 36 | >150 | 25.91 | 173.80 | 100 |
| 37 | 76.2 | 3.97 | 56.40 | 100 |
| 38 | 141.88 | 4.78 | 121.90 | 100 |
| 39 | 70.44 | 0.10 | 196.60 | 100 |
| 40 | 108.24 | 0.17 | 62.90 | 100 |
| 41 | 101.21 | 0.20 | 117.30 | 100 |
| 42 | 98.83 | 0.04 | 117.10 | 100 |
| 43 | 68.36 | 7.53 | 129.10 | 100 |
| 44 | 81.082 | 14.60 | 119.70 | 100 |
| 45 | 101.68 | 0.04 | 78.60 | 100 |

| **Table S2. Correlation between lymphocyte subsets and Tg levels before RAIT.** | | | | | |
| --- | --- | --- | --- | --- | --- |
| **Percentage** | **Spearman r** | **P value** | **Absolute numbers** | **Spearman r** | **P value** |
| **T%** | -0.129 | 0.397 | **T (cells/µL)** | 0.141 | 0.335 |
| **B%** | 0.068 | 0.656 | **B (cells/µL)** | 0.170 | 0.264 |
| **NK%** | 0.082 | 0.594 | **NK (cells/µL)** | 0.133 | 0.384 |
| **CD4^+^ T%** | -0.153 | 0.316 | **CD4^+^ T (cells/µL)** | 0.093 | 0.545 |
| **CD8^+^ T%** | -0.025 | 0.873 | **CD8^+^ T (cells/µL)** | 0.118 | 0.438 |
| **Th1%** | 0.084 | 0.584 | **Th1 (cells/µL)** | 0.125 | 0.413 |
| **Th2%** | 0.022 | 0.886 | **Th2 (cells/µL)** | 0.075 | 0.625 |
| **Th17%** | 0.125 | 0.413 | **Th17 (cells/µL)** | 0.148 | 0.331 |
| **Treg%** | -0.031 | 0.839 | **Treg (cells/µL)** | 0.067 | 0.661 |

*p<0.05, **p<0.01, ***p<0.001.

| **Table S3. Correlation between lymphocyte subsets and Tg levels at day 30 after RAIT.** | | | | | |
| --- | --- | --- | --- | --- | --- |
| **Percentage** | **Spearman r** | **P value** | **Absolute numbers** | **Spearman r** | **P value** |
| **T%** | -0.005 | 0.976 | **T (cells/µL)** | -0.014 | 0.929 |
| **B%** | 0.110 | 0.473 | **B (cells/µL)** | 0.073 | 0.635 |
| **NK%** | -0.033 | 0.831 | **NK (cells/µL)** | 0.020 | 0.898 |
| **CD4^+^ T%** | 0.013 | 0.932 | **CD4^+^ T (cells/µL)** | -0.004 | 0.979 |
| **CD8^+^ T%** | -0.036 | 0.816 | **CD8^+^ T (cells/µL)** | -0.031 | 0.842 |
| **Th1%** | 0.164 | 0.282 | **Th1 (cells/µL)** | 0.100 | 0.512 |
| **Th2%** | 0.161 | 0.289 | **Th2 (cells/µL)** | 0.081 | 0.596 |
| **Th17%** | 0.017 | 0.912 | **Th17 (cells/µL)** | -0.010 | 0.947 |
| **Treg%** | 0.056 | 0.714 | **Treg (cells/µL)** | 0.004 | 0.980 |

*p<0.05, **p<0.01, ***p<0.001.

| **Table S4. Correlation between lymphocyte subsets and Tg levels at day 90 after RAIT.** | | | | | |
| --- | --- | --- | --- | --- | --- |
| **Percentage** | **Spearman r** | **P value** | **Absolute numbers** | **Spearman r** | **P value** |
| **T%** | -0.265 | 0.078 | **T (cells/µL)** | 0.008 | 0.957 |
| **B%** | 0.064 | 0.677 | **B（cells/µL）** | 0.100 | 0.515 |
| **NK%** | 0.230 | 0.129 | **NK（cells/µL）** | 0.257 | 0.088 |
| **CD4^+^ T%** | 0.000 | 0.998 | **CD4^+^ T（cells/µL）** | 0.107 | 0.484 |
| **CD8^+^ T%** | -0.251 | 0.097 | **CD8^+^ T（cells/µL）** | -0.092 | 0.547 |
| **Th1%** | 0.012 | 0.936 | **Th1（cells/µL）** | 0.079 | 0.607 |
| **Th2%** | -0.021 | 0.891 | **Th2（cells/µL）** | -0.011 | 0.942 |
| **Th17%** | -0.013 | 0.935 | **Th17（cells/uL）** | 0.102 | 0.507 |
| **Treg%** | 0.050 | 0.747 | **Treg（cells/uL）** | 0.112 | 0.466 |

*p<0.05, **p<0.01, ***p<0.001.
